# Identifying Recrudescent *Plasmodium falciparum* in Treated Malaria Patients by Real-time PCR and High Resolution Melt Analysis of Genetic Diversity

**DOI:** 10.1038/s41598-018-28179-2

**Published:** 2018-07-04

**Authors:** Khalid B. Beshir, Nouhoum Diallo, Colin J. Sutherland

**Affiliations:** 1 Department of Immunology & Infection, Faculty of Infectious & Tropical Disease, London School of Hygiene & Tropical Medicine, London, United Kingdom; 2 Malaria Research and Training Centre (MRTC), Department of Epidemiology of Parasitic Diseases, Faculty of Pharmacy, University of Sciences, Techniques and Technologies of Bamako, Bamako, Mali; 3 Department of Clinical Parasitology, Hospital for Tropical Diseases, University College London Hospitals Foundation Trust, London, United Kingdom

## Abstract

Recurrent parasitaemia during follow up of clinical trials of antimalarial drug efficacy results from either recrudescence of parasites surviving treatment or from parasites newly emerging from the hepatic stage of infection. Nested PCR is used to distinguish these two possibilities and the technique is difficult to standardise. There is risk of both false positive and false negative results, leading to misclassification errors. The high-resolution melt (HRM) assay was developed with pairs of conserved primers targeting blocks of merozoite surface protein 1 and 2 (*msp1 and msp2*) genes, and polymorphisms were compared using sequence-confirmed *Plasmodium falciparum* DNA samples from laboratory isolates. In this study, the HRM dissociation profiles of *msp1* and *msp2* amplicons were determined and validated against parasite isolates from malaria patients. The *msp1 and msp2* profiles of both laboratory and clinical isolates were reproducibly differentiated by HRM. These rapid assays are performed in a closed-tube system, and so avoid cross-contamination while increasing throughput, which are two major advantages. The HRM assays offer significant gains in simplicity, speed and interpretation of results, and reduced analysis cost, for studies that require discrimination of parasite clones. Assay performance in large-scale studies utilizing DNA samples derived from filter-paper bloodspots should now be evaluated.

## Introduction

*Plasmodium falciparum* is the most virulent of the six *Plasmodium* species that cause malaria in humans, being responsible for high mortality and morbidity, particularly in Africa. Efforts to control and eliminate malaria have been hampered by the emergence of drug resistant parasites^1^, but our ability to track and control resistance is also hampered by the high genetic diversity of *P*. *falciparum*. Further, to accurately estimate true drug efficacy in clinical trials of antimalarial drugs, recurrent infections seen after drug treatment need to be identified either as new infections arising from the liver or as recrudescent parasites persisting from the original infection. A PCR-correction method is generally deployed to distinguish between the two. In addition, *ex vivo* and *in vitro* studies to identify drug resistant parasites in complex infections also need to identify the constituent parasite clones in an infection or isolate in order to accurately capture the emergence of minor clones in the subsequent growth of parasites^2,3^, as is also seen *in vivo*^4^. This requires the identification of clones in paired samples, before and after treatment, and determining whether they are the same clones^5^.

One tool commonly used to distinguish between newly emergent and recrudescent parasites is conventional nested-PCR and gel electrophoresis detection. In this genotyping method, the genes that code the surface antigen loci of merozoite surface protein 1 and 2 (*msp1*,*msp2*) and glutamate rich protein (*glurp*) are amplified using sequence specific primers in a nested-PCR^6^. The recurrent infection is categorized by comparing the size of polymorphic repeat regions in these genes before (day 0) and after treatment (day of failure). If fragment sizes differ, the recurrent infection is categorized as a new infection, but if the post-treatment analysis produces the same fragment sizes as before treatment, these are considered recrudescent parasites, implying that treatment failure has occurred. In multi-clonal infections, minority clones constituting a small proportion of the total biomass might fall below the detection limit of the genotyping method and be missed by PCR-based detection due to competition for primers or other constituents of the reaction mix by the more abundant clones^7^. This was resolved by genotyping at extra time points on day 1 and day 2 post-treatment as well as at the follow-up time-point prior to the day that microscopically detected parasite recurrence occurred^5,8^. Using this approach, studies of treated malaria patients in Kenya and Tanzania showed the presence of additional malaria clones, differing from those at day 0 and day of failure resulting in re-classification of many recurrent infections as recrudescent infections in these extended analyses^5,8,9^. However, this approach requires significantly more effort, and current methods for determining different clones such as nested-PCR, microsatellite and DNA sequencing are labour-intensive, time-consuming and are prone to contamination. In addition, gel electrophoresis-based methods discriminate clones based on size differences alone and cannot detect sequence variation. In this study, a real-time quantitative PCR (qPCR) was developed with (HRM) method analysis, which is sensitive to both amplicon length and base composition, to identify distinct *P*. *falciparum msp1* and *msp2* genotypes.

## Results

### Primer optimization and validity of the assay

Alignments of *msp1 and msp2* genes from 3D7, Dd2, 7G8, K1, FCR3, R033 and HB3 were assessed to design oligonucleotide primers that amplify the conserved regions (data not shown). To establish the specificity of the oligonucleotide primers and optimize the PCR conditions, 3D7 DNA obtained from laboratory-cultured parasites were initially used. Amplification of *msp1* resulted in one minor peak and the major peak was retained in melt curve analysis but only the major one in HRM normalized graph windows. The *msp2* genotyping of 3D7 DNA, however, unexpectedly produced two major melt peaks and HRM amplification curves each. It was first thought this resulted from the presence of two amplicons due to contamination. Further repeats of the msp1 and *msp2* HRM analyses with a different source of DNA again gave two amplification curves for msp2, and fractionation of PCR products by gel electrophoresis confirmed that. Both *msp1 and msp2* generated single band each of the predicted size. To rule out any inherent problems of the assays, we used uMELT software^10^ to predict the HRM curve of *msp1 and msp2* gene fragment sequence of 3D7. The software correctly predicted one major and one minor curve for *msp1* and two major curves for *msp2* suggesting that this pattern does indicate two amplicons, but rather it is due to a bimodal melt of the double stranded DNA (dsDNA) where the base composition if not uniform throughout. The dsDNA melts in transition with regions of the amplicon that are more stable (e.g., G/C rich) melting later. These stable regions maintain their dsDNA configuration until the temperature is high enough to cause melting of the stable region, resulting in a bimodal melt profile even though only one amplicon is produced in the reaction.

**Figure 1:**
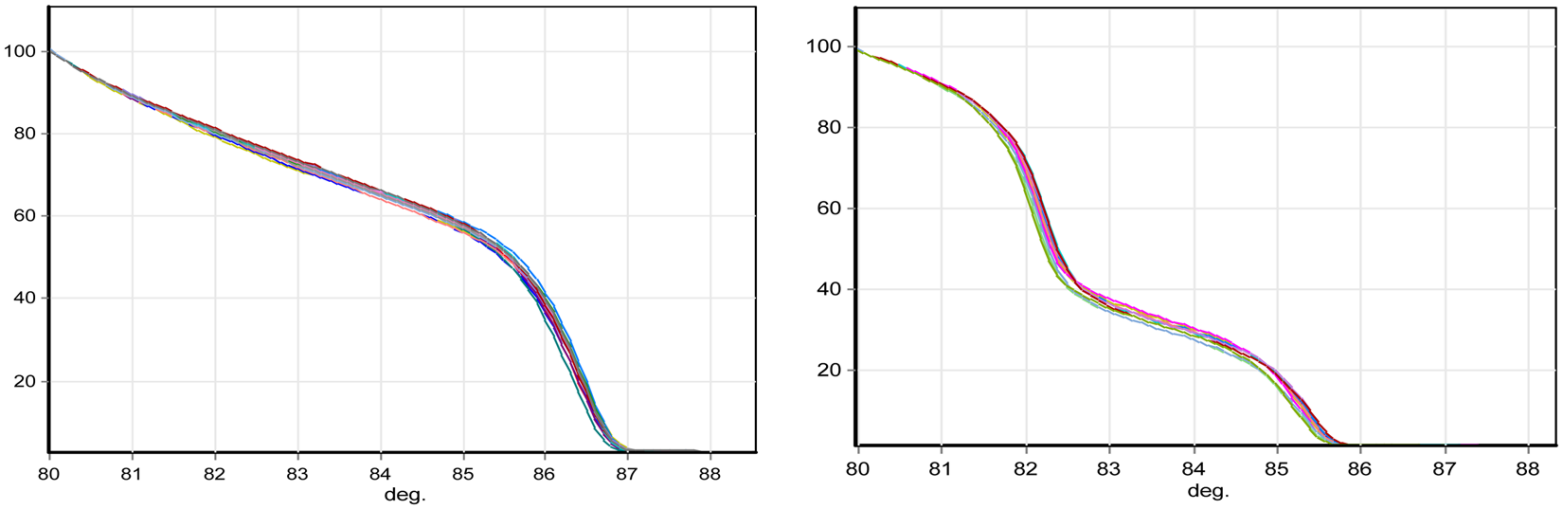
Normalized temperature-shifted HRM curves of *msp1* and *msp2* genes of *P*. *falciparum* 3D7 DNA. Each target was amplified in 35 tests with parasite DNA from a culture of 3D7 at 15 parasites per µl. The analysis software classified all samples as one genotype with (Genotype Confidence Percentage (GCP) of 71.9–99.5% for *msp1* and 71.8–99.3% for *msp2* genotype. Left: *msp1*. Right: *msp2*.

To determine the limit of detection of the *msp1* and *msp2* HRM assays, 3D7 DNA at different parasite densities (3–0.00003%) was amplified. The assay detected as low as 1.5 parasites per µl with GCP of 77.44% and 62.44% for *msp1* and *msp2*, respectively, relative to a 3D7 DNA sample at 1500 parasites per µl. GCP estimates increased to 92.32% and 78.41% for *msp1 and msp2* genotypes respectively when 15 parasites per µl (0.0003% parasitaemia) were used. We then chose the 3D7 DNA sample with 15 parasites per µl, which is closer to limit of detection of parasites by expert or reference laboratory microscopy, to determine the inter-assay coefficient of variation (CV). The mean ± SD inter-assay CV (n = 35) for *msp1 and msp2* was 93.15 ± 5.99 and 86.45 ± 9.47 respectively (Fig. 1).

**Figure 2:**
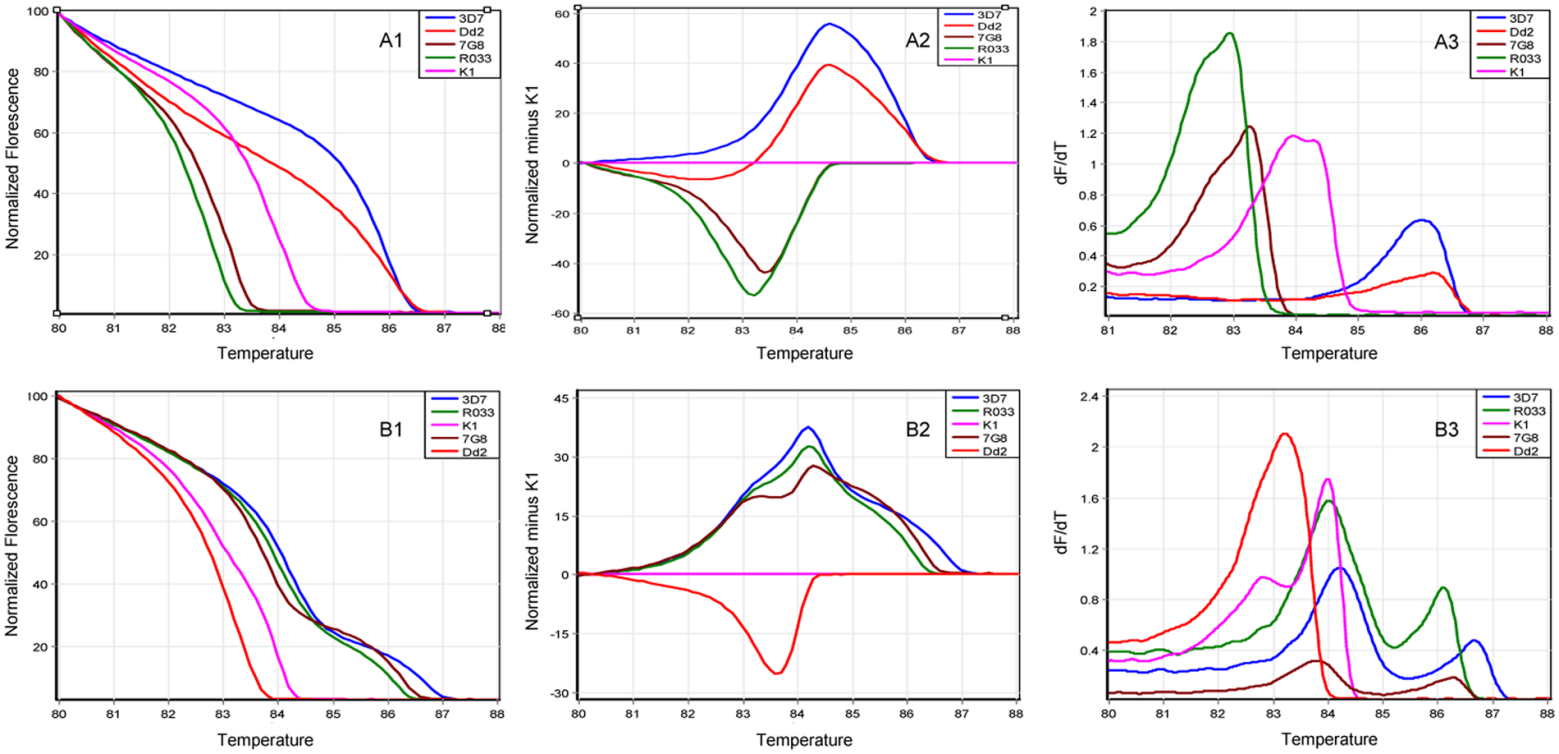
Normalized HRM analysis of *msp1* (**a1**–**3**) and *msp2* (**b1**–**3**) genotypes in 5 parasite lines. HRM amplification curves (**a1** and **b1**), difference curves (**A2** and **B2**) and melting curves (**A3** and **B3**) for 5 *P*. *falciparum* laboratory strains are shown. K1 is deployed as the comparator genotype in (**a2** and **b2**). dF/dT: negative derivative of change in florescence.

In an analysis of monoclonal parasite preparations, a distinct HRM amplification curve was produced for each of the five different clones (3D7, Dd2, 7G8, K1 and R033) using *msp1* and *msp2* genotypes (Fig. 2). DNA samples with 0.3%, 0.03% and 0.003% parasitaemia generated mean GCP of 76.8%, 78.2%, 99.3%, 95.5% and 88.9% for the 5 strains, respectively, when genotyped with *msp1* primers and mean GCP of 78.2%, 74.5%, 86.1%, 61.5% and 87.9%, when genotyped with *msp2* primers. The clones with lower melting temperature (Tm) (due to a shorter nucleotide sequence) such as 7G8, K1 and R033 in *msp1* genotype were less affected by parasitaemia difference compared to the clones with higher Tm (longer nucleotide sequence) such as 3D7 and Dd2 (data not shown). The minor peak observed in 3d7 *msp1* genotyping was also observed in all other laboratory isolates and was consequently excluded from HRM analysis.

### Detection of mixed laboratory clones

**Figure 3:**
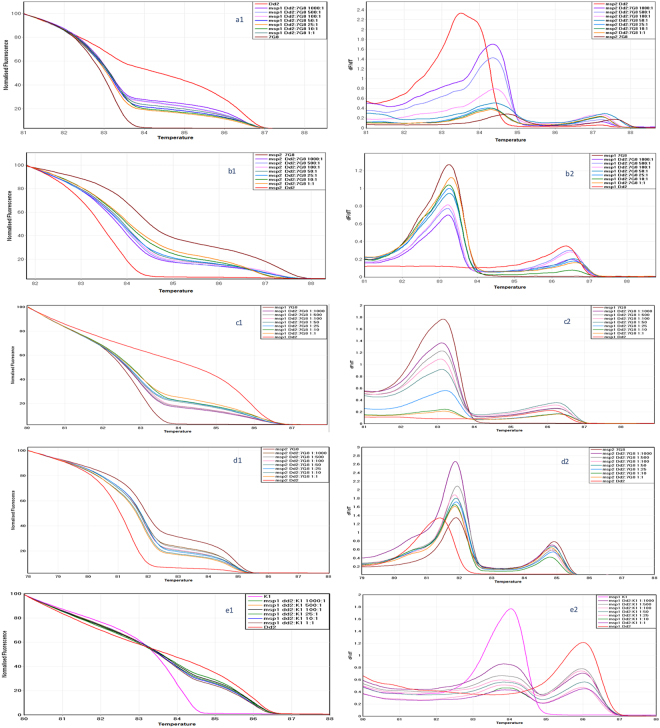
*msp1*, *msp2* HRM discrimination of artificial mixes of parasite clones at different ratios. Normalized temperature-shifted HRM amplification curves for *msp1* (**a1**,**c1** and **e1**) and *msp2* (**b1** and **d1**) and their corresponding melt curves (**a2**,**b2**,**c2**,**d2** and **e2**) are shown for Dd2, K1 and 7G8 laboratory lines, and mixes of two strains at the ratios shown.

To study the sensitivity of the assay when mixed clones are present in a sample, we mixed Dd2 and 7G8, and Dd2 and K1 in different ratios. As predicted, *msp1* genotype as well as the *msp2* genotype for Dd2 produced two distinct HRM curves when two clones were mixed (Fig. 3). The *msp2* genotype produced two HRM curves and three melt curves when two clones were present in the sample. These results have also be been confirmed in the melt curve analysis window (Table 1). If the parasitaemia of a particular laboratory strain in the mixture increases the HRM curve shifts toward the HRM curve of that particular strain. In all samples, the parasitaemia was adjusted to a maximum of 0.3% (15,000 parasites per µl) and minimum of 0.0003% (15 parasite per µl). Samples containing the same ratios of genotypes but different parasite density generally gave similar results but with smaller GCP. However, the higher the Tm value differences between mixed clones, the lower the GCP when calling the genotype similarity.

**Table 1:** Mean melting peak and mean GCP* of single and mixed laboratory strains.

Laboratory strain	Mix ratio	Genotype called by the software	*Msp1*		*Msp2*	
			Mean GCP *	Tm*	Mean GCP*	Tm*
3D7	—	3D7	99.3	86.0	99.9	84.0 and 86.5
Dd2	—	Dd2	97.5	86.4	99.7	83.50
7G8	—	7G8	99.7	83.3	99.7	84.3 and 87.3
K1	—	K1	99.9	84.0 and 84.3	99.7	82.8 and 83.8
R033	—	R033	99.9	82.9	99.5	84.3 and 87.3
Dd2:7G8	1:1	Dd2/7G8	99.6	83.2 and 86.5	98.7	83.9 and 86.9
	10:1	Dd2/7G8	91.8	83.2 and 86.5	74.4	83.9 and 86.9
	25:1	Dd2/7G8	88.3	83.2 and 86.5	68.4	83.9 and 86.9
	50:1	Dd2/7G8	76.5	83.3 and 86.6	62.7	83.9 and 86.9
	100:1	Dd2/7G8	73.9	83.2 and 86.5	62.4	83.9 and 86.9
	500:1	Dd2/7G8	63.15	83.2 and 86.5	59.5	83.9 and 86.9
	1000:1	Dd2/7G8	61.4	83.2 and 86.5	52.88	83.9 and 86.9
	1:1000	Dd2/7G8	99.3	83.2 and 86.3	56.3	81.9 and 84.9
	1:500	Dd2/7G8	62.8	83.2 and 86.3	61.2	81.9 and 84.9
	1:100	Dd2/7G8	73.2	83.2 and 86.3	64.5	81.9 and 84.9
	1:50	Dd2/7G8	78.2	83.3 and 86.3	70.2	81.9 and 84.9
	1:25	Dd2/7G8	89.2	83.2 and 86.3	73.6	81.9 and 84.9
	1:10	Dd2/7G8	91.2	83.2 and 86.3	78.5	81.9 and 84.9
	1:1	Dd2/7G8	99.3	83.2 and 86.3	97.9	81.9 and 84.9
	1:1	Dd2/K1	99.8	84.0 and 86.0	—	—
	10:1	Dd2/K1	93.9	84.0 and 86.0	—	—
	25:1	Dd2/K1	89.7	84.0 and 86.0	—	—
	50:1	Dd2/K1	86.5	84.0 and 86.0	—	—
	100:1	Dd2/K1	83.5	84.0 and 86.0	—	—
	500:1	Dd2/K1	73.2	84.0 and 86.0	—	—
	1000:1	Dd2/K1	71.4	84.0 and 86.0	—	—

*For samples with a single allele, the mean GCP estimates agreement between duplicate tests while the mean GCP for the mixtures is estimated from 2 mixtures of Dd2 and 7G8 at the ratio shown, relative to the 1:1 mixture.

**Tm: melting temperature; this is estimated by the rotor-gene 6000 analysis software (version 1.7). Mixture clones of Dd2 and K1 was not done as both belong to the same allelic family.

### Patient samples

**Figure 4:**
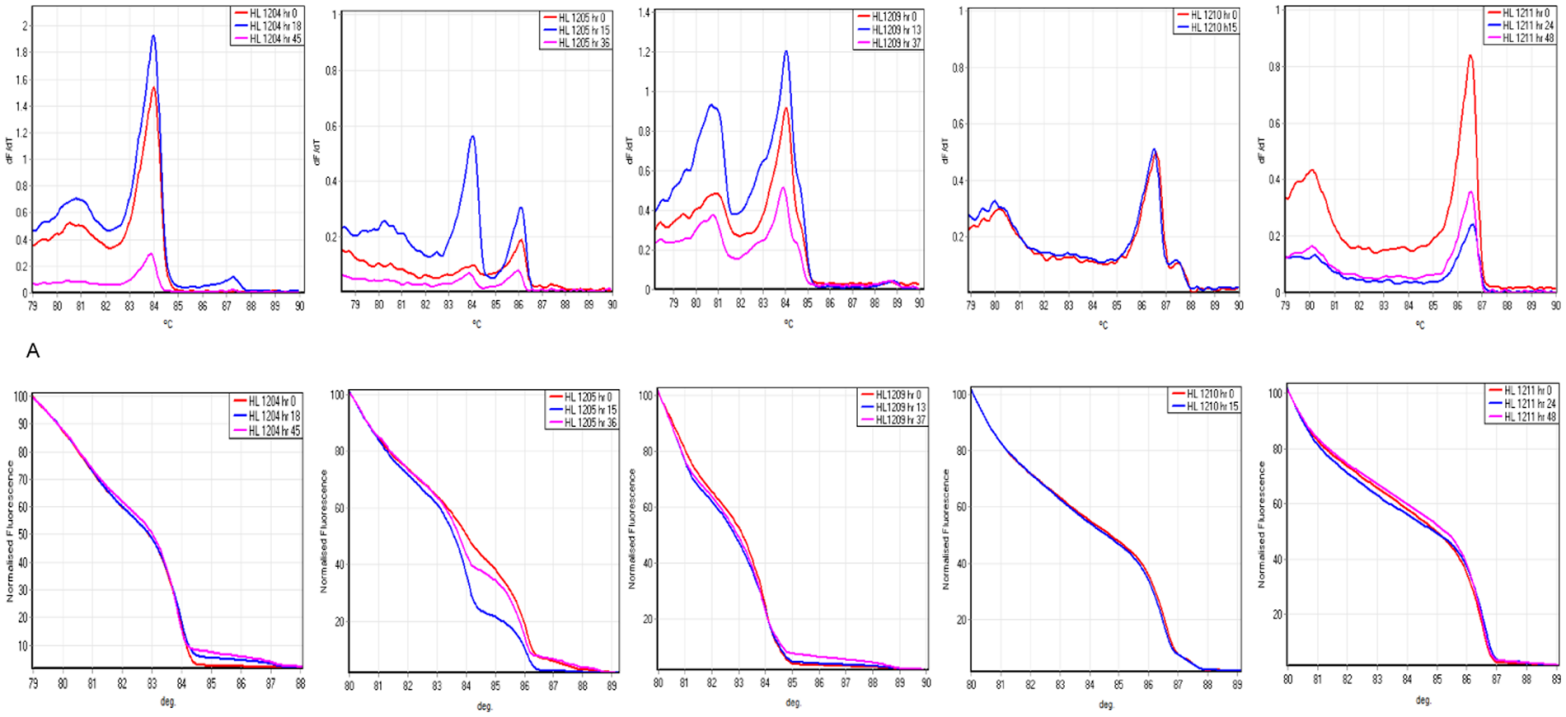
*msp1* HRM analysis of paired clinical samples from the same individuals. Melting amplification curves (top graphs) and normalized temperature-shifted HRM amplification curves (bottom graphs) of *msp1* genotypes in 5 patients sampled before (hr 0) and after treatment (hr 13–48). Differences in raw florescence signal between timepoints were observed, but didn’t affect HRM genotype calls after normalization. dF/dT, negative derivative of change in florescence and C°, temperature.

**Figure 5:**
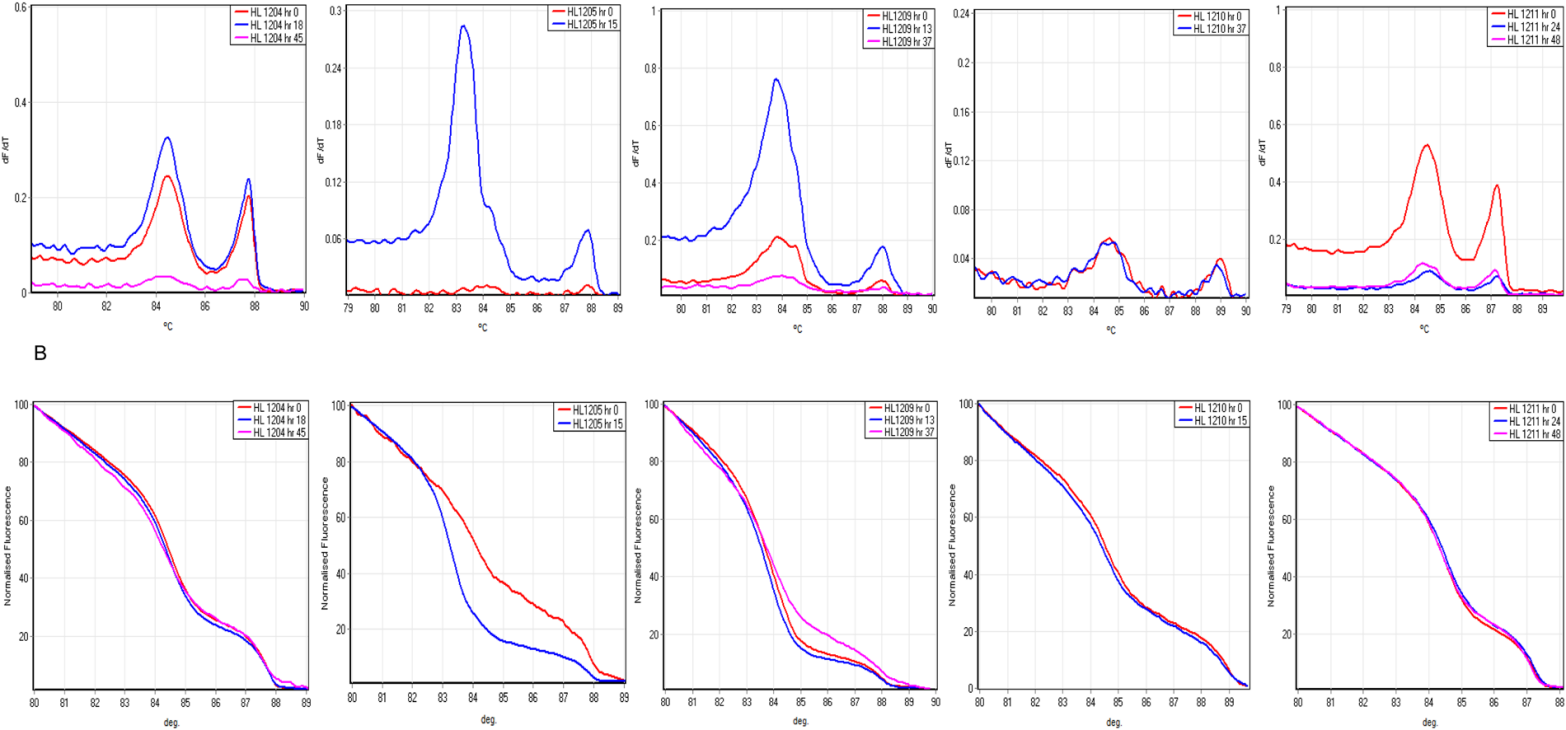
*msp2* HRM analysis of paired clinical samples from the same individuals. Melting amplification curves (top graphs) and normalized temperature-shifted HRM amplification curves (bottom graphs) of *msp2* genotypes in 5 patients sampled before and after treatment.

We genotyped paired pre- and post-treatment blood samples from five Hospital for Tropical Diseases (HTD) patients, and one additional unpaired pre-treatment sample. We investigated whether the assay accurately detects the number of clones in each sample and correctly classifies before and after treatment samples as the “same” or “different” infections. The HRM/melt curves generated by *msp1* and *msp2* genotypes for each patient are presented in Figs 4 and 5. With the exception of HL1210 and HL1211, all patient samples on post-treatment carried an extra HRM/melt curve when genotyped by *msp1* compared to corresponding pre-treatment samples (Fig. 4). Generally, the additional HRM peak in the post-treatment samples displayed lower fluorescence intensity compared to other peaks, suggesting a minor clone, which was below the detection limit of the assay in the pre-treatment samples. This difference was reflected in the genotype calling of the paired samples, where HL1204 and HL1209 were classified as the “same” infection with a genotype confidence percentage (GCP) of 78.6% and 62.3%, respectively, indicating moderate similarity to the pre-treatment sample, compared to low similarity for HL1205 (<50%) (Table 2). HL1210 and HL1211 produced very similar HRM/melt curves for pre and post treatment samples, and as a result, their GCP were 90.6% and 93.1% respectively, showing much greater similarity between the two time points than the patients with an extra clone in post-treatment samples. On the other hand, *msp2* genotyping produced the same HRM/melt curve in all paired patients samples with the exception of HL1205, where an extra curve was observed (Fig. 5). Consequently, the GCP was much higher (87.5–97.5%) compared to their corresponding GCP of patient samples obtained by *msp1* genotyping. Paired DNA samples of HL1205 patient had GCP of less than 50% confirming the GCP obtained by *msp1* genotyping.

**Table 2:** Genotype calling of paired clinical samples using GCP* of *msp1* and *msp2*.

Patient ID	Time after treatment	Genotype called by the software compared to hr 0	*Msp1* Confidence % relative to hr 0	*Msp2* Confidence % relative to hr 0
HL 1204	hr 0	HL1204	99.53	99.41
	hr 18	HL1204	82.17	82.95
	hr 45	HL1204	59.52	55.65
HL 1205	hr 0	HL1205	98.59	90.75
	hr 15	Variation	<50	<50
	hr 36	HL1205	70.27	Negative
HL 1209	hr 0	HL1209	99.77	98.72
	hr 13	HL1209	56.35	69.63
	hr 37	HL1209	57.80	<50
HL 1210	hr 0	HL1210	94.75	98.52
	hr 15	HL1210	73.23	74.35
	hr 40	Negative	NA	NA
HL 1211	hr 0	HL1211	99.83	95.89
	hr 24	HL1211	65.63	88.57
	hr 48	HL1211	77.51	94.24

All clinical samples after treatment had the same genotype compared to their corresponding samples before treatment (hr 0) samples with a GCP of more than 50%. Exceptions are HL1205 and HL1209, where both had a post-treatment genotype different from the pre-treatment in one or more loci. A second time-point after treatment was included for comparison purposes and in order to further evaluate the performance of *msp1* and *msp2* HRM genotyping for low parasitaemia samples. *GCP, Genotype Confidence Percentage, percentage of similarity of post-treatment sample to its corresponding pre-treatment sample.

The allelic family of the DBS field samples was determined relative to the HRM melt curve of the positive controls with known allelic family (RO33 for *msp1* or Dd2 for *msp2* and K1 and 3D7 for both), which represent the three different HRM melt curve observed in the initial validation experiment. Any sample within 0.2 °C degree of the sample with known HRM melt curve belongs to the same clone (Supplementary Fig. S1A–C).

All DBS field samples collected on day 0 showed the same HRM melt curve (the same Tm value) using both *msp1* and *msp2* assays compared to the samples collected 7 days later (Fig. S1A–C). On the other hand, the day 14 sample of one child showed one less clone compared to day 0 and day 7 while the day 21 sample of another child had one different clone compared to day 0 and 7 (Supplementary Table S1). The *msp1* and *msp2* HRM melt curve results were similar to clones generated by gel-based electrophoresis. One sample (K011) on day 28 was negative by *msp1* but showed similar HRM melt curve on *msp2* though slightly different Tm was observed (84.2 on d0 and 83.8 on day 28). However, the normalized HRM curve classified the two samples as “different”. This is consistent with the gel-based PCR, which generated different clones on d0 (K1 and RO33) and d28 (K1 and MAD20) suggesting a possible new infection (Supplementary Fig. S2).

To confirm the above HRM melt curve analysis, a GCP analysis was performed on all field samples. Samples collected before treatment and 7 days after treatment were classified as the “same” with a mean GCP of 85% (64–99%) for *msp1* and 82% (61–99%) for *msp2* in all samples. Samples collected after day 7 (day 14, 21 and 28) were classified as “different” with a GCP of less than 50% relative to day 0 sample. This was in complete agreement with the gel-based PCR results (Supplementary Fig. S2).

### GCP cut-off value for distinguishing between recrudescence and new infection

When compared to DNA prepared from a 3D7 culture at 3% parasitaemia, DNA from the lowest parasitaemia 3D7 culture (0.0003%) produced GCP estimates of 77.44% and 62.44% for *msp1* and *msp2* respectively. When Dd2 and 7G8 were artificially mixed, DNA from a 10:1 to 1000:1 mixtures at 0.3% parasitaemia generated a GCP estimate for *msp1* and *msp2* of >50% in comparison with a 1:1 mixture at the same parasite density (Table 1). This GCP estimate is a better reflection of actual field isolates, and fits with the WHO definition of a new infection, which requires that all alleles, in one or all loci tested, should be different in both time-points. Therefore, we opted to use GCP of 50% as a cut off value to distinguish recrudescence from new infections. This cut-off value was applied for the paired pre- and post-treatment patient samples and accurately classified four of the five as the “same” infections with a satisfactory GCP (≥62.3%). This was expected as the paired samples were obtained before and 13–48 hrs after antimalarial treatment and new infections are not expected at this time.

### Genetic diversity study

**Figure 6:**
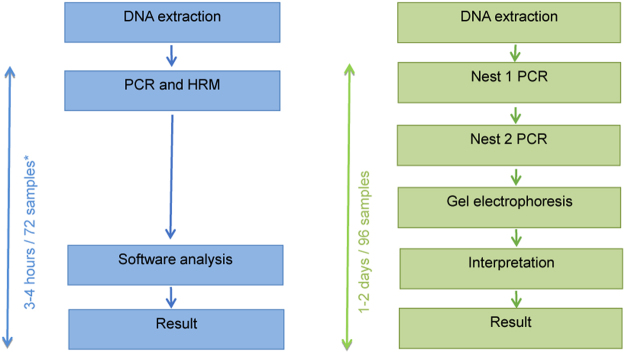
Benefits of *msp1* and *msp2* genotyping by real-time PCR and HRM. Summary of significant gains in simplicity, speed and interpretation of results from utilizing the qPCR-HRM genotyping approach compared to the conventional *msp1 and msp2* genotyping using gel electrophoresis. *3–4 hours per run (72 samples for rotor-gene and 96 samples for other platforms).

To assess the general relevance of the assay for applications requiring analysis of genetic diversity, the difference graph was used to cluster clones based on their similarities. Using the standard gel-based *msp1 and msp2* genotyping, parasite isolates are broadly classified into K1, MAD20 and R033, and FC and IC/3D7 sub family clones respectively. Since the Tm value for the K1 clone was found to lie in the middle of the range of values across all laboratory isolates tested, this clone was used as a calibrator and the software calculated the florescence signal of each clone relative to the signal of K1 and to generate a difference graph (Fig. 2). The approach correctly clustered the *msp2* clones into two groups, cone comprising 3D7, 7G8 and R033, and the second represented by Dd2. These groups correspond to IC/3D7 and FC sub family alleles originally described by Snounou and colleagues respectively^6,7^. The *msp1* difference graph clustered the clones into three groups containing 3D7, Dd2 (MAD20), 7G8 (R033). In gel-based discrimination, 3D7 and K1 are grouped together but in the HRM assay, they cluster into different groups, reflecting difference in the DNA sequence and length. Interestingly, the patient DNA samples HL1204 and HL1209, HL1205 and HL1212, and HL1210 and HL1211 clustered into 7G8/R033, Dd2 (MAD20) and 3D7 allele types respectively with GCP ranging from 42.6% to 78.2% when genotyped with *msp1*. When genotyped with *msp2* HL1204, HL1210 and HL1211 clustered into IC/3d7 sub family clones while HL1205, HL1209 and HL1212 clustered in both IC/3D7 and FC type clones suggesting that both alleles were present in the samples. This has been previously reported for these isolates using gel electrophoresis, where the samples contained more than 3 or more clones of FC type and more than 1 or more clones of IC/3D7 type^3^. Using HRM, the classification of parasite clones into clusters of sub-families takes 3–4 hrs compared to 1–2 days using PCR and gel-electrophoresis methods (Fig. 6).

## Discussion

Genotyping of the *msp1* and *msp2* loci has been deployed to assess multiplicity of infection, to evaluate strain-specific selection in vaccine efficacy studies, to estimate the genetic diversity of *P*. *falciparum* in population genetic studies, and to distinguish between recrudescent and novel genotypes in recurrent infections occurring in participants treated for malaria in the course of antimalarial drug efficacy trials. These studies have most commonly used PCR and gel electrophoresis, for discrimination among different *msp1* and *msp2* alleles^6,11^. The specificity of PCR- identification of recrudescent infections can be improved by deploying multiple post-treatment time points^5,9^. However, if using the nested-PCR and gel-electrophoresis method, this approach is laborious, resource intensive and can cause difficulties in interpretation. The current study reports the development of real-time PCR based HRM technology for msp1 and msp2 genotyping a new tool for *in vivo* and *in vitro* drug and vaccine efficacy studies. HRM analysis is a closed tube approach for fast, accurate, high throughput genotyping and provides a simple method for PCR-correction. This eliminates the limitations of gel electrophoresis and subjective characterisation of the different alleles. Unlike electrophoresis, HRM can distinguish alleles based on differences in base composition, sequence and amplicon size, delivers quantitative estimate of allele similarity (GCP), and has been successfully evaluated for detection of drug resistance malaria parasites^12^.

The *msp1 and msp2* HRM assays correctly discriminated alleles from five different single-clone laboratory isolates, those of polyclonal DNA samples derived from whole blood and DBS. The assays detected densities as low as 1.5 parasites per µl accurately, suggesting that the method is as sensitive as the nested-PCR electrophoresis method. However, the mixed clones evaluated were artificially prepared and the ability of HRM assays to precisely estimate the probability of missed alleles in natural field isolates remains to be determined. This was clear when paired patient samples were genotyped and analysed using HRM, where at least one allele was missed in two patient samples compared to the nested-PCR and gel electrophoresis method^3^. It is not clear whether this is due to an intrinsic sensitivity difference in detecting minor clones or the detection of false positive alleles by nested-PCR and gel electrophoresis, possibly due to amplicon contamination. HRM assays avoid contamination and risk of PCR product carry-over as the method is non-nested and uses a closed-tube readout.

Both *msp1 and msp2* HRM genotyping identified one or more extra alleles in post-treatment samples from some of the patients. This was also observed previously when the same samples were genotyped by PCR and gel electrophoresis, suggesting that this is a general observation regardless of the PCR-based genotyping methods used^3,13^, best explained by changes in the relative abundance of the constituent circulating clones *in vivo* before and after treatment^5,8^. In polyclonal infections, these changes in relative abundance mean certain genotypes may remain undetected at one time point, but recur later leading to false classification. These minority clones are missed by PCR-based detection methods due to competition for primers or other constituents of the reaction mix by the more abundant clones^7^. In a study in Papua New Guinea, detection of minor clones decreased with an increase in multiplicity of infection, suggesting the dominance of major clones among PCR amplicons detected^14^. A similar study carried out in Uganda reported a higher probability of mixed infections in high transmission areas, and this affected classification of treatment outcomes^15^. A slow-clearing minority variant present but not detected at admission, could cause recrudescence but would be identified in the recurrent sample as a new infection, rather than recrudescence of parasite genotypes present at the time of treatment. This has recently been demonstrated in a multiplicity of infection study to detect and quantify the markers of antimalarial drug resistance^16^. Therefore, the failure to detect minor alleles at day 0 is likely to lead to misclassification of some infections^17^. The data in this study and previous reports^3,5,8^ demonstrate that inclusion of the additional time points day 1, day 2 and day before failure, in addition to pre-treatment and day of failure, improve specificity when determining the complexity and origins of recurrent infections.

One other strength of the HRM assay is that the clones are discriminated based not only on length but also sequence differences and this feature allows the HRM assay to overcome the resolution limitation observed in the gel electrophoresis-based methods^6^. This is particularly important in high transmission settings, where two distinct clones may generate PCR products with the same size but which differ in sequence. These will be indistinguishable by gel electrophoresis. Others have attempted to resolve this by classifying any pair of samples that contain matched and unmatched alleles as new infections^18^, but this can clearly lead to underestimation of recrudescence infections. WHO/MMV guideline states that, in treated malaria patients, “ a ‘re-infection’ [i.e. new infections] is a subsequent occurring parasitaemia in which all the alleles in parasites from the post-treatment sample are different from those in the admission sample, for one or more loci tested”^19^. This definition seeks to minimize misclassification error and maximize accuracy of classification.

One of the shortcomings of the HRM assays is that some of the *msp1* and *msp2* alleles generate more than one melting peak, due to the inherent two-phased nature their DNA melting profile. For distinguishing recrudescence from new infections, this shortcoming can be overcome as the HRM amplification curves of the samples before and after treatment are compared with each other, and any additional peak due to the inherent nature of DNA melting will be reflected in both samples. The interpretation of those melting peaks will be more challenging if the genotyping purpose is to study parasite diversity and complexity of infection, although we have shown that this can be overcome by including known comparators for both *msp1* and *msp2* genotyping. Measurement of the amplification signal of each clone relative to these comparators thus permits classification of allelic variants into clusters.

In addition to drug and vaccine efficacy studies, HRM genotyping can be applied in studies of parasite biology – for example, to assess parasite cloning experiments, and in monitoring the identity, integrity and clonality of propagated parasite lines, if necessary direct from cryo-preserved material. The HRM method can also be deployed as tool for *in vitro* drug sensitivity studies, particularly for polyclonal parasite isolate in which a subset of genotypes preferentially survive *in vitro* cultures, as this often reflect survival in *in vivo* therapeutic efficacy studies^20^. Our data warrant the large-scale evaluation of the performance of the real-time PCR and HRM approach on DNA samples derived from filter paper bloodspots collected in field studies.

## Materials and Methods

### *Plasmodium falciparum* DNA strains and field samples

Parasite DNA from malaria cases treated at the Hospital for Tropical Diseases (HTD), London, UK was utilized for this study. Clinical isolates were obtained from six patients: pre-treatment and post-treatment samples were available for patients HL1204, HL1205, HL1209, HL1210 and HL1211 and only pre-treatment for patient HL1212. Details of the patient history, parasitaemia and treatment have previously been published^3^. Eighteen paired dried blood spot (DBS) samples were available to validate the HRM on DBS field samples. For 15 children, day 0 (before treatment) and day 7 (7 days after treatment) were available. For two children, day 0 and day 14 or day 28 samples were available and for one child, day 0, 7 and 21 samples were available. The samples were collected between January and July 2014 from asymptomatic children aged 5 to 12 years attending schools near Mbita, Kenya, as part of a study on mosquito behaviour and odor profile of malaria-infected individuals. Study site, sample collection and other details of the study has already been published^21,22^. Culture adapted laboratory isolates 3D7, Dd2, 7G8, K1, R033 and D10 were obtained from the Malaria Research and Reference Reagent Repository (http://MR4.org). Parasite cultures were tightly synchronized as ring-stage trophozoites to stimulate infected peripheral blood *in vivo*.

### PCR

New primer sets were designed to amplify block 1 of *msp1* and block 3 of *msp2* genes. Pairs of oligonucleotide primers for *msp1* (*Msp1*HRM_F: TAGAAGATGCAGTATTGACAGGT and *Msp1*HRM_R: CAGCGTAAGATTTAGCATCTGAATC) and msp2 (*Msp2*HRM_F: AGCAACACATTCATAAACAATGCT) and *Msp2*HRM_R: TCCATGTTGTCCTGTACCTTTATTC) flanking the target regions were designed with the PCR amplicon expected to yield 346 bp and 518 bp respectively. The *msp1* primers are conserved in the commonly used isolates K1, MAD20 and Ro33, and the *msp2* primers in FC27 and 3D7/IC respectively. Amplification of DNA was performed in a 25 µl reaction volume on a Rotor-gene 6000 thermal cycler (QIAGEN, Germany). The reaction mixture contained 5 µl of extracted genomic DNA, 200 nM of each primer, 3 mM of MgCl2, 300 nM of each dNTP, 5 µM SYTO 9 green florescence nucleic acid stain (Invitrogene), 1X NH4 reaction buffer (Bioline, UK) and 1 U Taq polymerase (Biolione, UK). PCR conditions were one cycle of 94 °C for 2 min, 40 cycles of 94 °C for 15 sec, 54 °C for 20 sec and 72 °C for 40 sec. To verify the specificity of the primer sets, PCR products were detected by gel electrophoresis of 10 µl from each reaction on 2% agarose gels. Gel-based PCR assays targeting *msp1* and *msp2* genes were amplified using previously reported methods^6^ and clone similarity between time points was carried out using previously published approaches^5,18^. Gels were made and run in TBE buffer (sigma-Aldrich, UK) and 5ul of loading buffer (Bioline, UK) were added to each sample prior to electrophoresis.

### Optimization and validation

The specificity of the *msp1* and *msp2* primers was established by running gel-electrophoresis of the PCR products. The sensitivity and limit of detection of the assay was estimated using a five-fold serial dilution of 3D7 in blood starting at 3% parasitaemia. The sensitivity to detect clones in mixed infection was estimated using a mixture of Dd2 and 7G8, and Dd2 and K1 *Plasmodium falciparum* in culture medium at 5% haematocrite in a ratio of 1:1, 10:1, 25:1, 50:1, 100:1, 500:1 and 1000:1, and Dd2:7G8 in a ratio of 1:1, 10:1, 25:1, 50:1, 100:1, 500:1 and 1000:1 with a minimum parasitaemia of 15 parasites per µl. The reproducibility of the *msp1* and *msp2* HRM assays was determined by carrying out 35 replicate tests of 15 parasites per µl of 3D7 strain.

### HRM curve acquisition

QPCR HRM analysis was performed in a Rotor-gene 6000 thermal cycler (Corbett Life Science). In order to determine the optimal melting temperature for differentiation of *P*. *falciparum* clones, the PCR products were subjected to a ramping of 0.1 °C s^−1^ between 75 °C and 90 °C. All specimens were performed in duplicate and their melting profiles were analysed using Rotor-gene 6000 software (version 1.7.87) and the HRM algorithm provided. Visual examination of the melt curves data from a panel of laboratory isolates across a gradient of different temperature ramp increments was performed to select the optimum temperature in which most laboratory isolates show distinct conventional melt curves.

### Genotyping analysis

For genotype analysis, temperature-shifted and normalized amplification curves were used in in the HRM analysis. Normalization regions of 82.0–82.5 and 88.0–88.5 were used as a standard but were modified for some clinical samples depending on the melting temperature peaks observed. The threshold in the melting curve analysis was adjusted to ensure that all positive samples generate interpretable dF/dT profiles and melt peak estimates. A fixed threshold was not deployed as the florescence signal amplitude varied across samples. The Genotype Confidence Percentage (GCP) is a value attributed to each genotype/allele compared with the genotype of a calibrator, with a value of 100% indicating an exact match. For clinical samples, the pre-treatment sample (hr 0) was a calibrator.

### Clonal and cluster analysis

Melt curve analysis was used to verify the presence of multiple clones in each parasite isolate or culture. Clone similarity was signified by assigning names of known genotypes (strains) to samples with similar melting temperature to the known genotypes. PCR amplicons from five different *P*. *falciparum* laboratory isolates were subjected to HRM curve analysis. The difference-curve graph was used to classify the different *msp1* and *msp2* clones into clusters based on florescence signal difference relative to K1 (reference strain).

### Analysis methods

Validation of each result was assessed using C_T_ value, end-point florescence level and amplification efficiency. The real-time data were analysed using different modules available in the Rotor-gene software. A sample was re-analysed if its C_T_ value was ≥30, or if it had lower amplification end point compared to the majority plots, or if amplification efficiency differ from other reactions or fell below an amplification value of 1.4 (2 = 100% amplification efficiency). The reproducibility of the *msp1* and *msp2* assays was measured by calculating the coefficient of variation (mean ± standard deviation) of GCP of replicate tests.

### Availability of data and material

All culture-adapted parasite lines described have been deposited in the European Malaria Reagent Repository (http://www.malariaresearch.eu/) and are freely available to researchers. All data generated or analysed during this study are included in this published article (and its Supplementary Information files).

### Ethics approval and consent to participate

All research was performed in accordance with LHSTM relevant guidelines. Voluntary informed written consent and travel history was obtained from patients presenting with malaria to the HTD, or the Accident and Emergency Department of University College London Hospitals (UCLH). Approval for the study was obtained from the Research Ethics Committee of the University College London Hospitals (Application number: 07/Q0505/60), and include in their manuscript a statement confirming that informed consent was obtained from all participants and/or their legal guardians

## Electronic supplementary material

Dataset 1
