# Supplementary material for: Identifying Recrudescent Plasmodium falciparum in Treated Malaria Patients by Real-time PCR and High Resolution Melt Analysis of Genetic Diversity
Source: Sci Rep. 2018 Jul 4;8:10097. doi: 10.1038/s41598-018-28179-2 (PMC6031682; doi:10.1038/s41598-018-28179-2)

Identifying Recrudescent *Plasmodium falciparum* in Treated Malaria Patients by Real-time PCR and High Resolution Melt Analysis of Genetic Diversity

Khalid B Beshir[1*], Nouhoum Diallo[2], and Colin J. Sutherland[1, 3]

1. Department of Immunology & Infection, Faculty of Infectious & Tropical Disease, London School of Hygiene & Tropical Medicine.
2. Malaria Research and Training Centre (MRTC), Department of Epidemiology of Parasitic Diseases, Faculty of Pharmacy, University of Sciences, Techniques and Technologies of Bamako, Mali.
3. Department of Clinical Parasitology, Hospital for Tropical Diseases, University College London Hospitals Foundation Trust

* Address correspondence to Khalid B Beshir, Department of Immunology and Infection, Faculty of Infectious & Tropical Disease, London School of Hygiene & Tropical Medicine. Keppel Street, London WC1E 7HT, United Kingdom. Email: Khalid.Beshir@lshtm.ac.uk

Key words: HRM, *msp1*, *msp2*, recrudescence, re-infection

Suplementary material

Figure S1. Determination of clones from dried bloodspot samples for *msp1* (A1) and *msp2* (A2) relative to positive controls; melting amplification curves for msp1 (B1) and msp2 (C1) and normalized temperature-shifted HRM amplification curves for msp1 (B2) and msp2 (C2) of genotypes in 18 kenya asymptomatic children sampled before (hr 0) and after treatment (day 7, 14, 21 or 28).

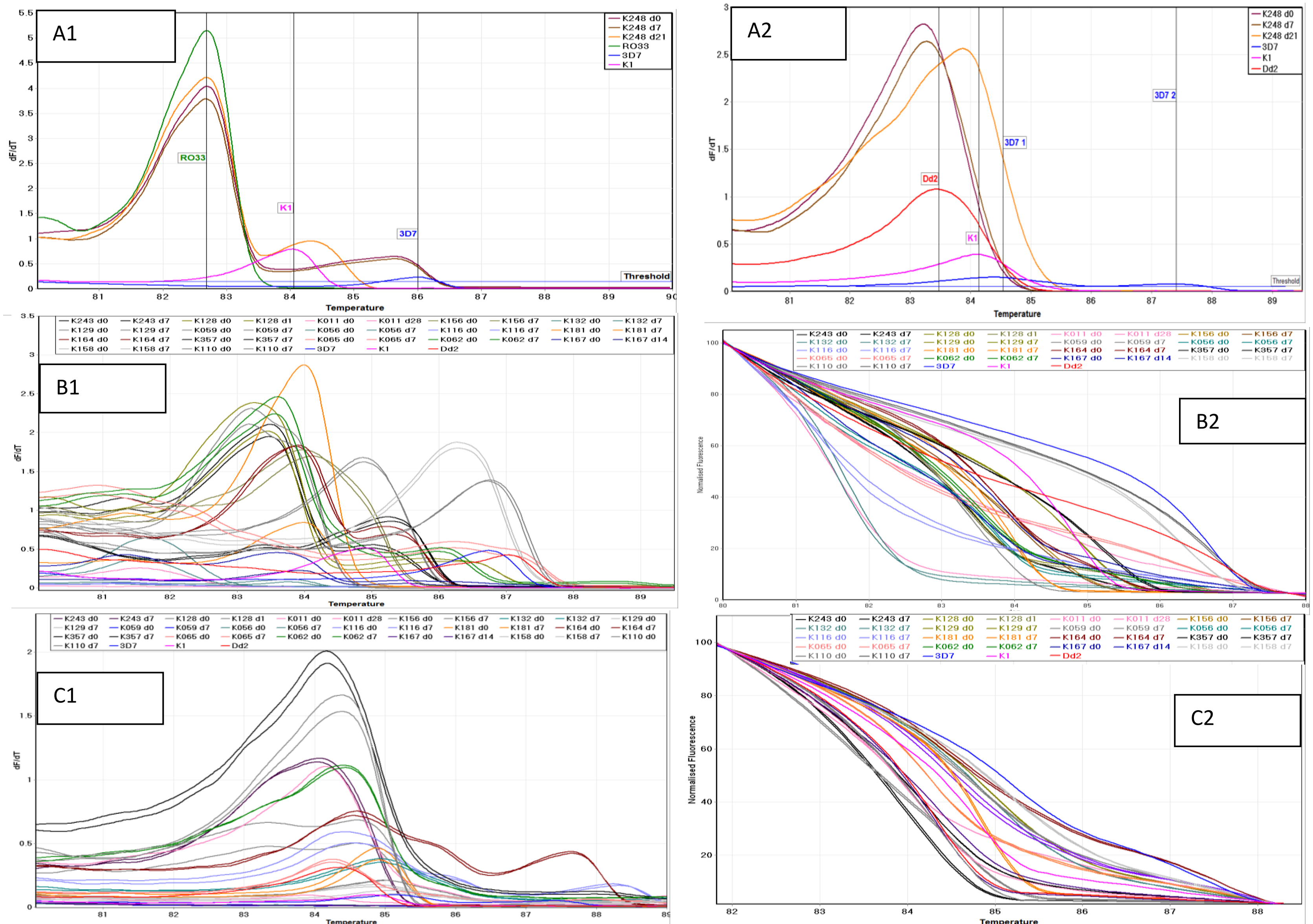

Table S1: **Clones and allelic family of field dried bloodspot samples generated by gel-based PCR and HRM genotyping using *msp1* and *msp2* assays**. The table shows six laboratory strains and 18-paired samples. *Msp1* and *msp2* HRM melt curves for the samples was determined by the software after threshold adjustment (Supplementary Fig S1 A) and the allelic family was determined relative to the laboratory strains with known clones.

Lab strain Patient ID	Gel electrophoresis Allelic family		*Genotype called by the HRM software Allelic family (lab strain)*		***Msp1* HRM melting curve**	***Msp2* HRM melting curve**
	**Msp1 (base pair)**	**Msp2 (base pair)**	***Msp1***	***Msp2***		
3D7	K1	IC/3D7	K1	IC3D7	86.0	85.30 and 87.5
7G8	RO33	IC/3D7	RO33	IC3D7	83.3	84.3 and 87.3
K1	K1	FC27	K1	FC27	84.0 & 84.8	82.8 and 84.8
RO33	RO33	IC3D7	RO33	IC3D7	83.2	84.3 and 87.3
Dd2	K1	FC27	Dd2	FC27	86.5	83.5
D10	MAD20	FC27	MAD20	FC27	81.6	83.5
K011 d0<br>K011 d28	K1 & RO33<br>K1 & MAD20	FC27<br>NEG	Dd2<br>NEG	FC27 (Dd2)<br>FC27 (Dd2)	81.6<br>NEG	84.2<br>83.8
K059 d0<br>K059 d7	RO33<br>RO33	FC27<br>FC27	RO33<br>RO33	FC27 (K1)<br>FC27 (K1)	83.2 & 83.8<br>83.2 & 83.8	84.6<br>84.6
K056 d0<br>K056 d7	K1<br>K1	FC27<br>FC27	7G8<br>7G8	FC27 (K1)<br>FC27 (K1)	83.8<br>83.8	84.0<br>84.0
K062 d0<br>K062 d7	K1<br>NEG	NEG<br>FC27	7G8 & 3D7<br>7G8 & 3D7	FC27 (Dd2)<br>FC27 (Dd2)	83.5 & 86.0<br>83.5 & 86.0	84.5<br>84.5
K065 d0<br>K065 d7	RO33 & K1<br>RO33 & K1	FC27<br>FC27	K1 & 3D7<br>K1 & 3D7	FC27 (Dd2)<br>FC27 (Dd2)	86.0 & 87.2<br>86.0 & 87.2	84.5<br>84.5
K110 d0<br>K110 d7	K1<br>K1	IC/3D7<br>IC/3D7	3D7<br>3D7	FC27 (Dd2) & IC/3D7 (3D7)<br>FC27 (Dd2) & IC/3D7 (3D7)	86.7<br>86.7	84.3, 85.5 & 88<br>84.3, 85.5 & 88

Lab strain Patient ID	**Gel electrophoresis** **Allelic family**		***Genotype called by the HRM software*** ***Allelic family (lab strain)***		***Msp1* HRM melting curve**	***Msp2* HRM melting curve**
	**Msp1 (base pair)**	**Msp2 (base pair)**	***Msp1***	***Msp2***		
K116 d0<br>K116 d7	MAD20<br>MAD20	IC/3D7 & FC27<br>FC27	RO33<br>RO33	FC27 (Dd2) & IC/3D7 (3D7)<br>FC27 (Dd2) & IC/3D7 (3D7)	81.4<br>81.4	84.5,85.5 & 88<br>84.5,85.5 & 88
K128 d0<br>K128 d1	RO33 & K1<br>RO33 & K1	FC27<br>FC27	RO33 & K1<br>RO33 & K1	FC27 (Dd2 & K1)<br>FC27 (Dd2 & K1)	83.5 & 86<br>83.5 & 86	83.4 & 84.5<br>83.4 & 84.5
K132 d0<br>K132 d7	RO33<br>NEG	IC/3D7 (2)<br>IC/3D7 (2)	RO33<br>RO33	IC/3D7 (3D7)<br>IC/3D7 (3D7)	82.5<br>82.7	85.0 & 89.0<br>85.0 & 89.0
K129 d0<br>K129 d7	K1<br>K1	FC27 & IC/3D7<br>FC27 & IC/3D7 (2)	K1<br>K1	FC27 (K1) &IC/3D7 (3D7)<br>FC27 (K1) &IC/3D7 (3D7)	84.8<br>84.8	84.6, 85.8 & 87.9<br>84.6, 85.8 & 87.9
K158 d0<br>K158 d7	RO33<br>RO33	FC27<br>NEG	3D7<br>3D7	FC27 (K1)<br>FC27 (K1)	86.3<br>86.3	84.8<br>84.8
K164 d0<br>K164 d7	K1(200, 240)<br>K1(200, 240)	IC/3D7<br>FC27 (250,300)	K1 & 3D7<br>K1 & 3D7	FC27 (K1) IC/3D7 (3D7)<br>FC27 (K1) IC/3D7 (3D7)	83.8 & 85.5<br>83.8 and 85.5	84.5, 85.5 & 87.5<br>84.5, 85.5 & 87.5
K167 d0<br>K167 d14	RO33 (220 & 300)<br>RO33 (220 & 300)	FC27<br>FC27	RO33, 7G8 & K1<br>7G8 & K1	FC27 (Dd2)<br>IC/3D7	81.3, 83.5 & 85.5<br>83.9 & 85.5	83.8<br>85.3 & 87.3
K156 d0<br>K156 d7	K1<br>NEG	FC27<br>FC27	D10 & 7G8<br>7G8	FC27 (K1)<br>FC27 (K1)	81.5 &84.1<br>84.1	84.5<br>84.5
K181 d0<br>K181 d7	K1<br>K1	FC27<br>FC27	MAD20 & 7G8<br>MAD20 & 7G8	FC27 (K1)<br>FC27 (K1)	81.2 & 84<br>81.2 & 84	84.5<br>84.5
K243 d0<br>K243 d7	RO33/K1<br>RO33, K1 (200,240) & MAD20	FC27<br>FC27	MAD20,RO33&K1<br>MAD20,RO33&K1	FC27 (Dd2)<br>FC27 (Dd2)	81.5, 83.2 &85.0<br>81.5, 83.2 &85.0	84.0<br>84.0
K251 d0<br>K251 d7	NEG<br>NEG	FC27<br>FC27	RO33 & 3D7<br>NEG	FC27 (Dd2)<br>FC27 (Dd2)	82.4, 83.37 & 85.2	84.3
K248 d0<br>K248 d7<br>K248 D21	K1 & RO33 (200, 250)<br>K1 & RO33 (200, 250<br>K1 (200,220,300)	FC27<br>FC27<br>FC27	K1 & 3D7<br>K1 & 3D7<br>K1 & RO33	FC27 (Dd2)<br>FC27 (Dd2)<br>FC27 (K1)	82.7 &85.9<br>82.7 &85.9<br>82.7 & 84.4	83.2<br>83.2<br>83.9
K357 d0<br>K357 d7	RO33 & K1<br>RO33 & K1	FC27<br>FC27	RO33 & K1<br>RO33 & K1	FC27 (Dd2)<br>FC27 (Dd2)	83.4 & 85.2<br>83.4 & 85.2	84.3<br>84.3

Figure S2: **Amplicon product of *msp1* PCR (K1, RO33 and MAD20) and *msp2* (FC27 and IC/3D7) of dried bloodspot samples on gel-electrophoresis**. Msp1 and Msp2 Allelic family of paired samples (day 0 and day 7 or 14 or 21 or 28) of 18 Kenyan asymptomatic children.

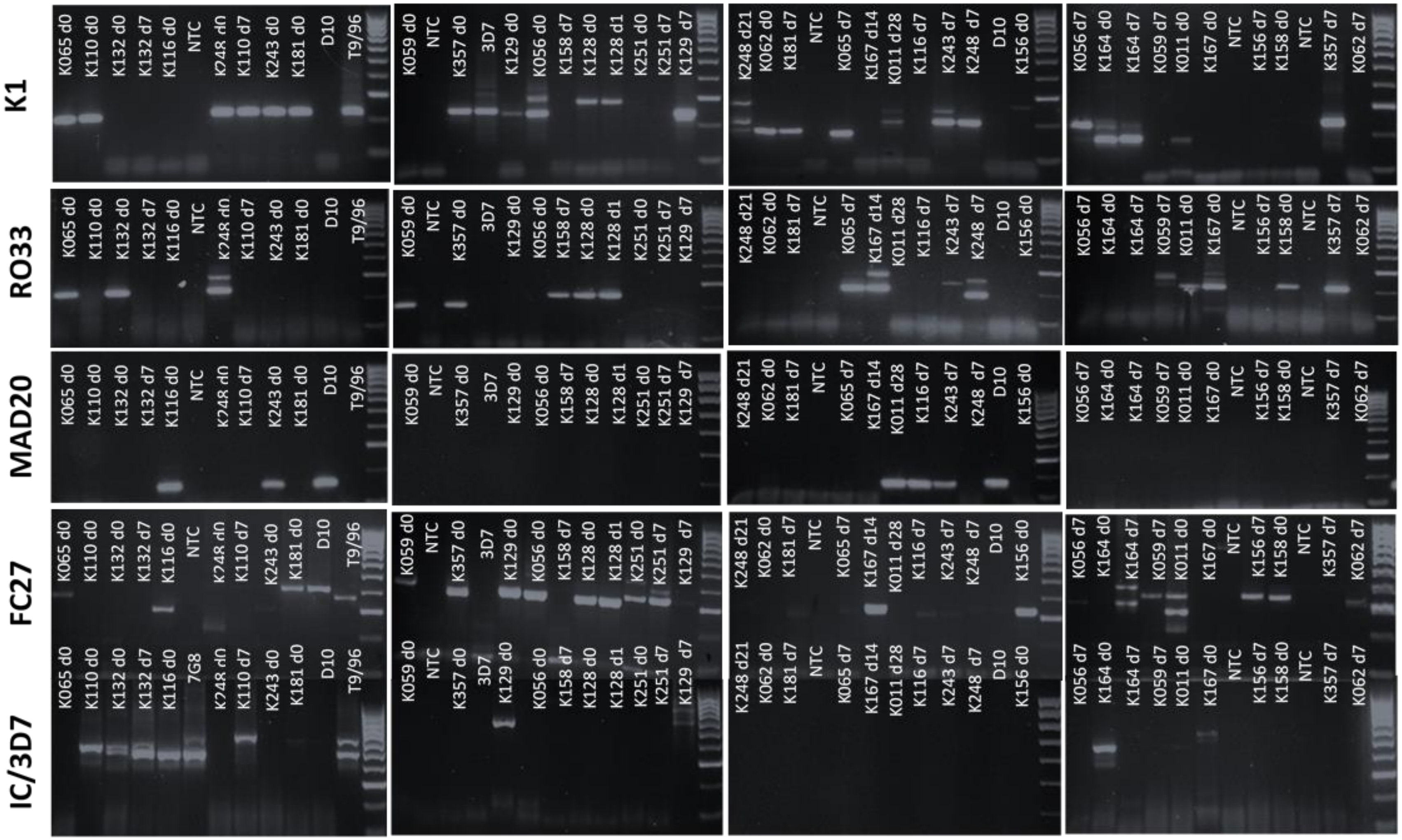

Supplement: Supplementary file 1 — Dataset 1 [file 41598_2018_28179_MOESM1_ESM.pdf]
